# Rebuilding vascular access: from the viewpoint of mechanics and materials

**DOI:** 10.3389/fbioe.2024.1448186

**Published:** 2024-09-04

**Authors:** Aurora Battistella, Morgan Linger, Anh Thy Nguyen, David Madukwe, Prabir Roy-Chaudhury, Wei Tan

**Affiliations:** ^1^ Paul M. Rady Mechanical Engineering, University of Colorado at Boulder, Boulder, CO, United States; ^2^ Department of Biomedical Engineering, University of Colorado at Boulder, Boulder, CO, United States; ^3^ Department of Medicine, University of North Carolina Kidney Center, Chapel Hill, NC, United States; ^4^ WG (Bill) Hefner VA Medical Center, Salisbury, NC, United States

**Keywords:** vascular access, dialysis, biomechanics factors, geometric design, arteriovenous graft (AVG), arteriovenous fistula (AVF), catheter

## Abstract

This review presents a comprehensive analysis of vascular access in hemodialysis, focusing on the current modalities, their associated challenges, and recent technological advancements. It closely examines the status of three primary types of vascular access: arteriovenous fistulas, arteriovenous grafts, and central venous catheters. The review delves into the complications and pathologies associated with these access types, emphasizing the mechanobiology-related pathogenesis of arteriovenous access. Furthermore, it explores recent clinical trials, biomaterials, and device innovations, highlighting novel pharmaceutical approaches, advanced materials, device designs, and cutting-edge technologies aimed at enhancing the efficacy, safety, and longevity of vascular access in hemodialysis. This synthesis of current knowledge and emerging trends underscores the dynamic evolution of vascular access strategies and their critical role in improving patient care in hemodialysis.

## 1 Introduction

The prevalence of end-stage kidney disease (ESKD) is rising alarmingly worldwide, particularly as populations with diabetes and the elderly expand (Thurlow et al., 2021). The US Renal Data System (USRDS) 2018 annual report reported over 700,000 prevalent cases of treated kidney failure in 2016. At a global level, the number of patients with treated kidney failure is expected to increase from 2.6 million in 2010 to 5.4 million in 2030 (Lawson et al., 2020; McCullough et al., 2019). This surge presents a significant public health challenge; in 2015 alone, 1.2 million deaths were attributed to kidney failure, and projections suggest a potential increase of 29%–68% by 2030 (Thurlow et al., 2021). Hemodialysis has remained the lifesaving and most common treatment for individuals with ESKD (Swaminathan et al., 2012). The effectiveness of this treatment critically depends on functional vascular access, which facilitates repeated blood transfer from the patient’s circulatory system to the dialysis machine (Almasri et al., 2016). The economic burden is also critical, in the United States in 2010, total Medicare spending on kidney replacement therapy was $34 billion (Lawson et al., 2020; Swaminathan et al., 2012). However, the extensive complications and frequent dysfunctions associated with vascular access highlight a pressing clinical need to address these challenges (Almasri et al., 2016).

This review begins with an assessment of the primary vascular access options for hemodialysis: arteriovenous fistulas (AVFs), arteriovenous grafts (AVGs), and central venous catheters (CVCs). The analysis provides insights into their distinct roles, advantages, and limitations, underscoring their significance in patient care and healthcare outcomes in managing ESKD. Subsequently, the focus shifts to the intricacies and challenges inherent in these arteriovenous access types. The discussion delves deeply into complications and explores the unique pathogenesis associated with arteriovenous access, highlighting the importance of structural and mechanical designs.

Advancing into recent developments, the review examines clinical trials and device innovations that are shaping the future of vascular access in hemodialysis. It sheds light on promising pharmaceutical interventions, novel biomaterials, and advanced technological applications poised to enhance the functionality and durability of vascular access methods.

The review aims to provide a holistic understanding of the current state and future directions in vascular access for hemodialysis, emphasizing the need for ongoing innovation to enhance patient outcomes and quality of life for individuals with chronic kidney disease.

## 2 Current state of vascular access

**FIGURE 1:**
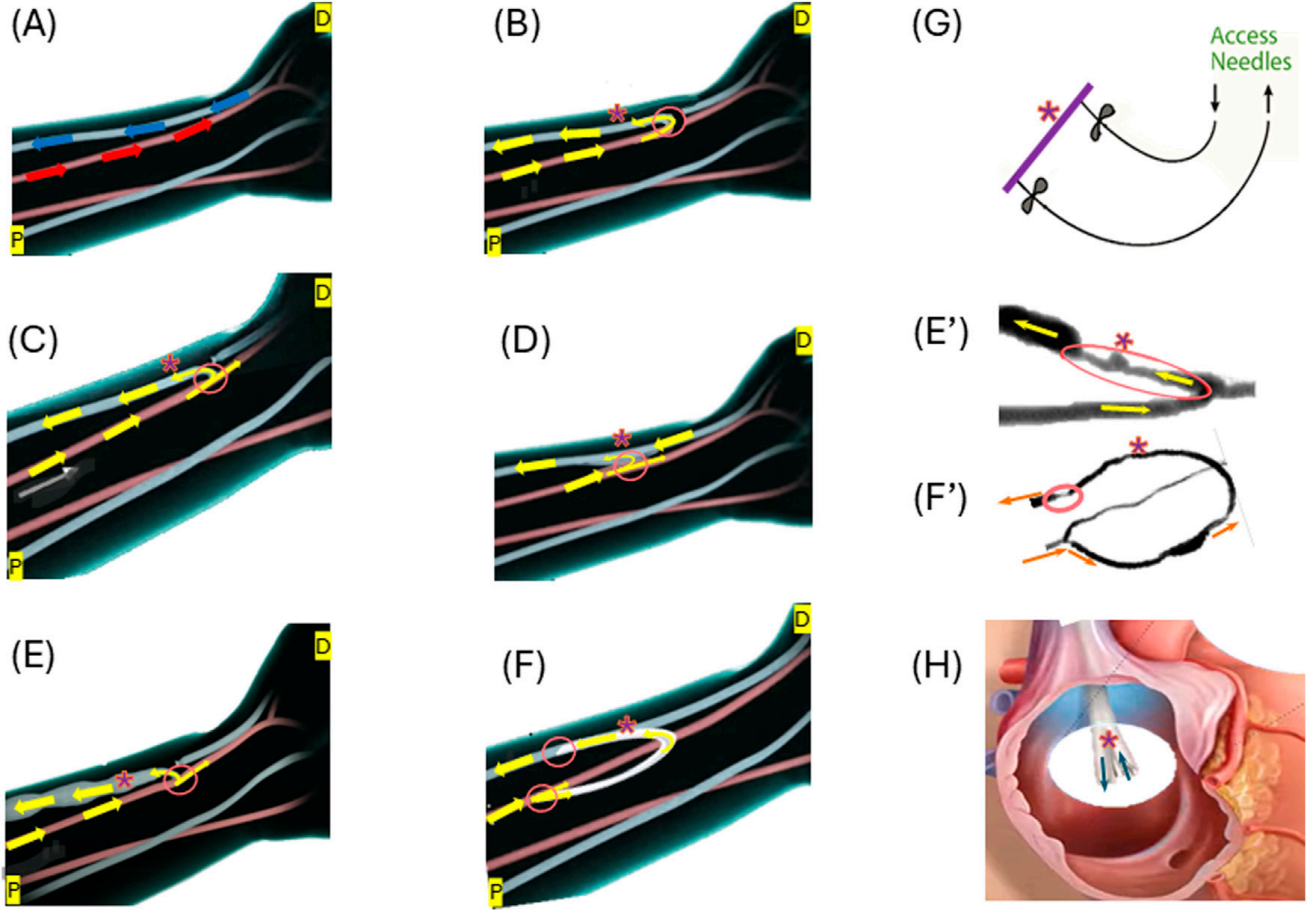
Illustrations of the three forms of vascular access: **(A)** Normal flow in the artery (red) and vein (blue) at a possible access location; **(B)** End-to-end anastomosis for AVF creation; **(C)** Side-to-end anastomosis for AVF creation; **(D)** Side-to-side anastomosis for AVF creation; **(E)** Matured side-end AVF; **(E’)** Stenosed AVF; **(F)** AVG formation; **(F’)** Stenosed AVG; **(G)** Needle access to arteriovenous vascular access; **(H)** An example of CVC–BD Pristine™ catheter. Arrows illustrate the flow directions. “*” indicate the access (AVF, AVG or CVC) for needles to access for dialysis. Circled areas illustrate anastomosis. Some illustrations were modified from images shown on the website of www.ypo.education.

The choice of vascular access is a pivotal consideration in hemodialysis treatment for ESKD patients, directly influencing treatment efficacy, patient comfort, and overall outcomes (Murea et al., 2019). The three primary types of vascular access (possible access location illustrated in Figure 1A) include AVF (Figure 1C), AVG (Figure 1F), and CVC (Figure 1H), as illustrated in Figure 1.

AVFs, a connection between the artery and the vein (Figure 1C), allow the patient’s blood vessel to “mature” to accommodate the high hemodialysis flow (Figure 1E) (Murea et al., 2019). Many medical communities consider them the optimal approach for hemodialysis access as they are the most durable and are associated with the fewest complications (Bylsma et al., 2017; Hafeez et al., 2023). During the AVF maturation process, the venous segment changes in diameter to increase the vessel blood flow, so that it can support hemodialysis. AVFs fail to mature because of a combination of neointimal hyperplasia and a lack of outward expansion (remodeling) (Remuzzi and Bozzetto, 2017). However, hemodialysis patients often have weakened veins that can not necessarily handle the increased blood flow required for successful treatment. The maturation process for an AVF is not only lengthy ranging from 4–6 weeks to 4 months or longer but also likely to fail (Li et al., 2018; Asif et al., 2006; Huber et al., 2021) reported the maturation rates of 67% at 6 months and 76% at 12 months after creation for 602 participants with nearly one-third requiring an intervention to facilitate maturation. With a failure rate anywhere between 28%–53% (Abreu, 2022), AVFs are far from a perfect option.

AVGs (Figure 1F) are an alternative to AVFs and tend to fail more often than AVFs (Franco, 2021), when a patient’s blood vessels are unsuitable for fistula creation or after multiple fistula failures (Murea et al., 2019). AVGs typically comprise synthetic materials like polytetrafluoroethylene (PTFE) or tissue-derived substances. They serve as a conduit between an artery and a vein (Figure 1F). AVGs have a considerable risk of thrombosis, with more than 50% of thrombosing within 12 months of creation (Hudson et al., 2019). The primary (unassisted) patency rates at 1 year range from 23% to 41%, and the secondary patency rates at 2 years range from 54% to 60% (Dixon et al., 2009; Li et al., 2024).

Major challenges of using AVFs or AVGs include neo-intimal hyperplasia (IH) often occurring in the vein, which leads to stenosis and ultimately access failure (Hudson et al., 2019). Examples of IH in AVFs and AVGs are shown in Figures 1E’, F’, respectively.

CVCs (Figure 1F) are currently limited to the use of short-term or temporary vascular access in hemodialysis when other options like AVF or AVG are not feasible. They are typically inserted into a large vein in the neck or chest (Lawson et al., 2020). The high risk associated with CVCs stems from their susceptibility to complications such as infections, thrombosis, and stenosis.

Table 1 provides a comparison of the three access forms. The current state of vascular access reflects a balance between the need for effective and long-lasting access against the challenges of patient-specific conditions and potential complications. Future innovations in materials and techniques should enhance the functionality and longevity of each vascular access form, as the choice of access type is highly individualized depending on various factors.

**TABLE 1:** Summary of three types of vascular access in hemodialysis.

Access type	Advantages	Challenges/Limitations	Clinical uses	Patient outcomes/Success rates	Citation
AVF	High patency rates, lower complication rates	- Lengthy maturation process - High failure rates of maturation - Around 55% usability within 4 months in the US	Preferred if patient’s vasculature is suitable	- Infection rate: 0.5%–1.5% perpatient-year - High primary unassisted patency rate: e.g., 57% and 71% for female and male patients after 5 years - Less likely to be abandoned	Asif et al. (2006), Li et al. (2018), Lawson et al. (2020), Hafeez et al. (2023), Liu (2023)
AVG	Suitable for patients with unsuitable vessels for AVF, reliable access	- Prone to neointimal hyperplasia - Complications at graft-vein anastomosis	Used when AVFs are not viable	- Infection rate: 13% - Primary patency at 2 years ∼40%, Secondary patency ∼60%	Hudson et al. (2019), Murea et al. (2019), Lawson et al. (2020), Halbert et al. (2020)
CVC	Immediate access, temporary solution	High rates of infection, thrombosis, and vein stenosis	Used when other options are not feasible	- Primary patency failure: 91% within the first year - Highest infection and complication rates among the three	Lawson et al. (2020)

## 3 Biomechanics and mechanobiological factors

**FIGURE 2:**
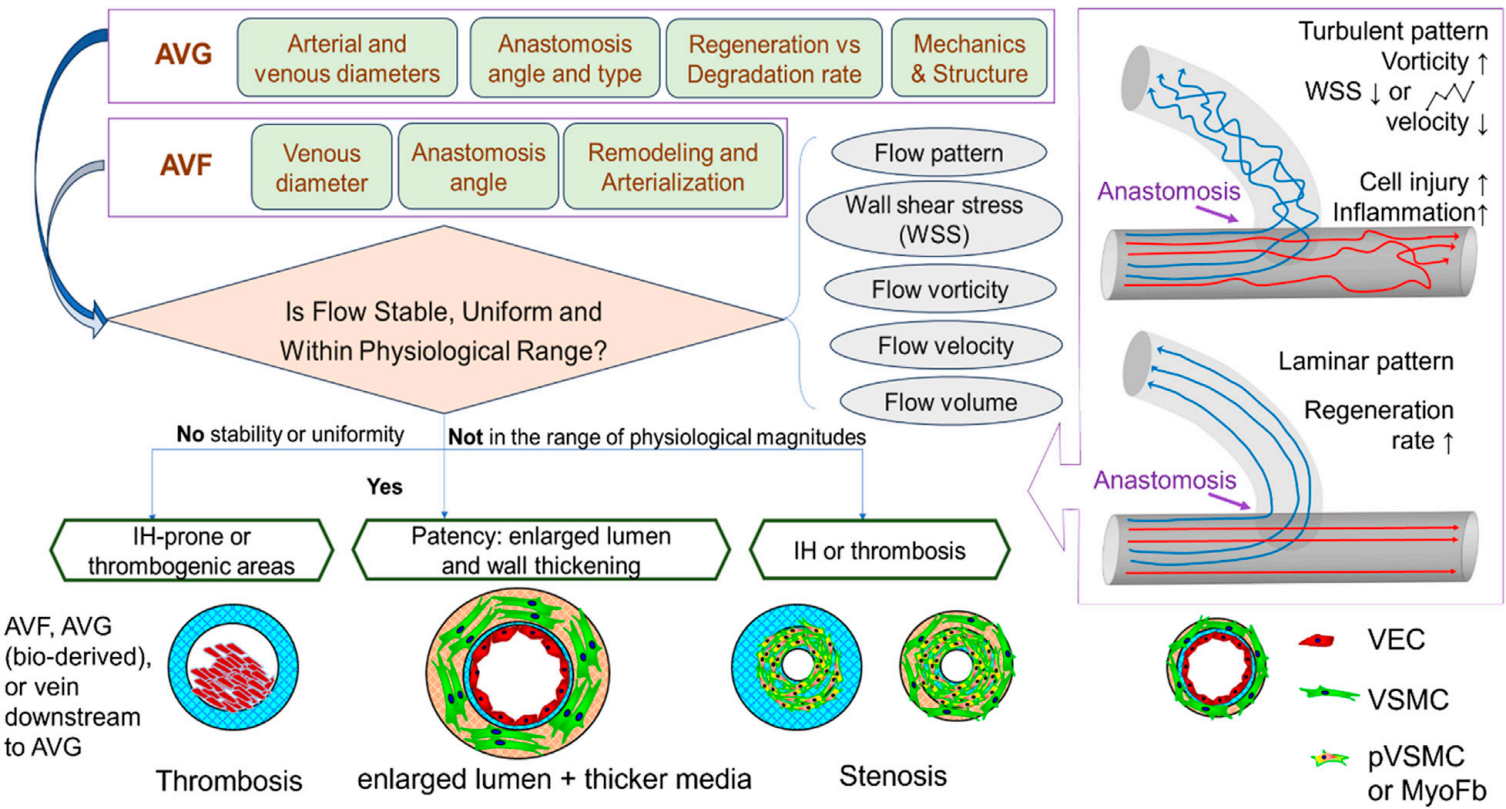
Illustrations of the determinants to and consequences of flow in the dialysis access. VEC, vascular endothelial cell; VSMC, vascular smooth muscle cell; PVSMC, proliferative VSMC; MyoFb, myofibroblast-like cell. VECs form the inner lining of blood vessels and play a crucial role in maintaining vessel function, regulating blood flow, and preventing clot formation. VSMCs provide structural support and help regulate vessel diameter.

This section reviews complications that jeopardize the patency of arteriovenous access. It highlights the role of flow around the vein close to the anastomosis, while briefly discussing other initiating factors such as cannulation-related complications. As summarized in Figure 2, structural and mechanical parameters are crucial in determining the success of an AVF or AVG for proper hemodialysis access. The success of vascular access relies on considerations of intricate mechanobiology from flow conditions such as wall shear stress (WSS) down to cellular mechanisms including reactions of vascular endothelial cells (VEC) and smooth muscle cells (VSMC). Additionally, a unique challenge for AVG is the host cells’ reaction and integration with the graft, which is associated with AVG design to be discussed in the next section.

Among pathological features causing AVF maturation failure and AVG or AVF abandonment, a central one is the IH development, which is characterized by excessive proliferation of VSMCs and/or vascular myofibroblasts and subsequent deposition of extracellular matrix (ECM) components within the vessel wall (Newby and Zaltsman, 2000). IH further leads to decreased blood flow, increased pressure, stenosis, and thrombosis, all of which result in maturation failure or vascular access abandonment.

### 3.1 Anastomosis angle

**FIGURE 3:**
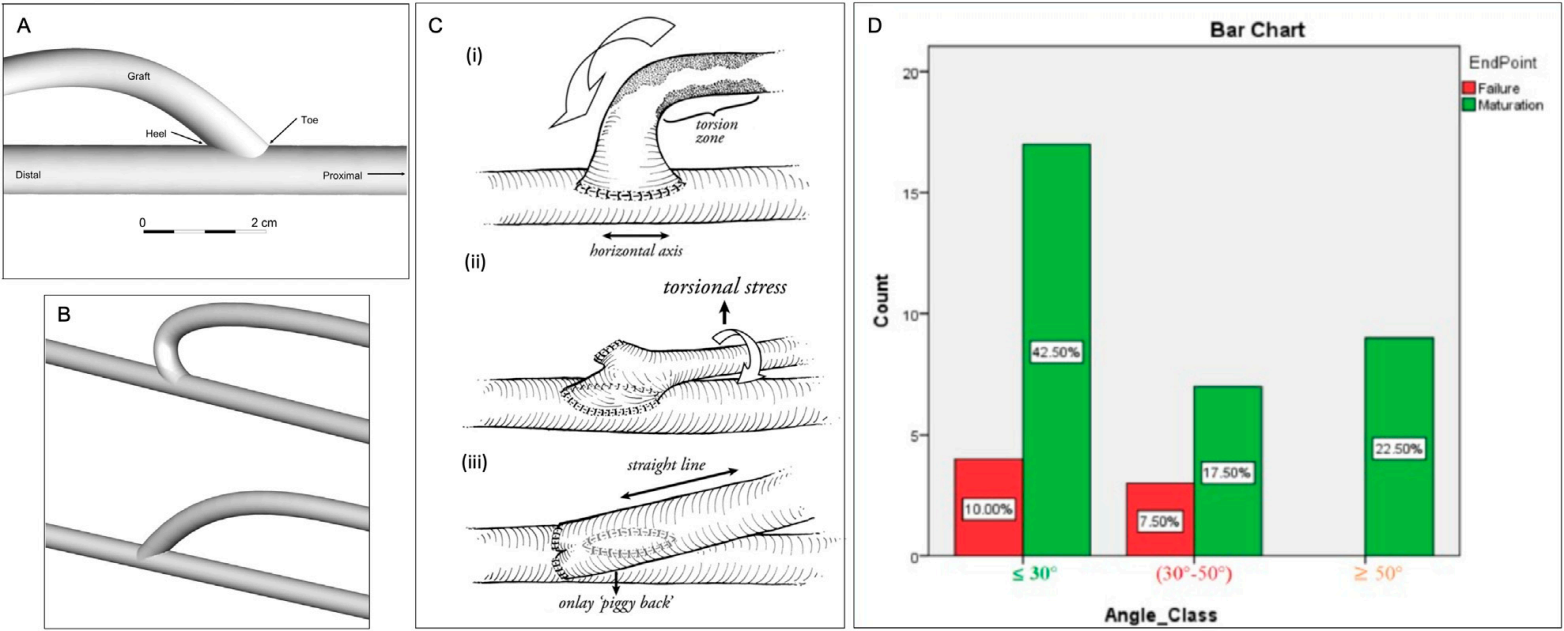
Anastomosis Angles. **(A)** Venous end of the graft demonstrating the beveled hood of the arteriovenous graft-to-vein anastomosis (Williams et al., 2021). **(B)** Geometries of modified (above) and standard (below) end-to-side arteriovenous fistula (AVF) configurations (Carrolll et al., 2019). **(C)** (i) torsional zone in end-to-side technique (ii) torsional component with side-to-side “SLOT” (iii) “Piggyback SLOT” technique (Bharat et al., 2012). **(D)** The crosstab analysis revealed that the least difference in AVF maturation and AVF failure rate occurred for anastomosis angles ≤30° (Rezapour et al., 2018).

Yang et al. (2020) investigated the impacts of anastomotic angle on blood flow through computational fluid dynamics (CFD) in models based on real patient images. They concluded that an anastomotic angle between 30 and 46.5° was optimal for the reduction of turbulent flow (Figure 3D), while anastomotic angles outside this range caused the shear stress to be concentrated in a single area of the AVF, or insufficient for AVF maturation (Grechy et al., 2017). Like AVF, the anastomosis angle largely influences the incidence of stenosis and thrombosis. Using a CFD model of an AVG, Williams et al. (2021) revealed an ideal anastomotic angle between 20 and 40°, while pathologically low and high shear rates occurred at angles below 20° and above 40°, respectively. Kim et al. (2021) further found that a high anastomosis angle led to unstable WSS–lower WSS area near the anastomosis but higher away from the anastomosis. Shembekar et al. (2022) determined that AVF should be formed at a 45° angle to avoid intimal hyperplasia (IH) if one believes that high WSS causes IH. Bharat et al. (2012) investigated a graft modification to reduce torsional stress, to prevent juxta-anastomotic stenosis in AVF (Figure 3C). Carrolll et al. (2019) developed a computational fluid dynamics model to examine the reduction of flow disturbances in a modified end-to-side AVF configuration. Their findings demonstrated that altering the anastomosis angle from 45° (Figure 3A) to 135° significantly decreased the flow disturbances typically associated with conventional, acute-angle, end-to-side AVFs (Figure 3B). Although many studies advocate for a 45° angle as the optimal configuration, this research suggests that a more gradual vein curvature could improve AVF maturation.

### 3.2 Anastomosis type

Related to anastomosis angle are the four anastomosis types, i.e. side-to-end (Figure 1C), end-to-end (Figure 1B), end-to-side, and side-to-side (Figure 1D). The most common type is end-to-side (ETS) anastomosis in which the end of the vein is connected to the artery. In a CFD model, side-to-side anastomosis provided a more uniform WSS distribution than 45° and 90° ETS anastomoses, thus avoiding areas of concentrated stresses prone to IH (Hull et al., 2013; Pike, 2021). Meta-analysis across a variety of studies also demonstrates better AVF patency at 12 months with side-to-side anastomosis with distal vein ligation compared to the standard end-to-side technique (Zhou and Wu, 2023). However, in clinical studies of cohorts of side-to-side and side-to-end anastomosis, there was no significant difference in patency outcomes (Mestres et al., 2019).

Thus, in idyllic models of anastomosis, side-to-side provides more uniform shear stress, however, the clinical practice does not show a significant difference in successful outcomes of side-to-side compared to ETS. There is no clear consensus on the optimal angle for anastomosis as it is highly dependent of the anastomosis type and the debated theory behind the causes of intimal hyperplasia. Speaking strictly for the classic ETS anastomosis, an angle of 45° allows for the most optimal WSS configuration.

### 3.3 Vessel selection

Through CFD, Krampf et al. (2021) found that the diameter and length of the inflow artery have a linear and inverse relationship, respectively, with flow volume delivered to the AVF. Thus, long or small-sized inflow arteries result in low flow volume to the AVF, which is in turn linked to stenosis (Abreu, 2022). However, high access flow with blood flow volume >1.5–2 L/min has negative impacts as well, such as an increase in left ventricular mass in addition to venous hypertension and heart failure/arrhythmia (Nojima and Motomiya, 2021). Thus, anastomotic selection should be made based on the patient’s specific needs for inflow, but larger diameter vessels are favored to deliver large flow volumes to AVF. Similarly, for AVGs, the arterial and venous diameters have also been linked to the AVG’s success. A CFD model indicated that arterial and venous diameters of above 4 mm and below 6 mm, respectively, along with an artery-to-vein ratio of 1:2 or 2:3, lead to lower shear stress and shear rate (Williams et al., 2021). Additionally, AVFs with a vein diameter below 2.15 mm and artery diameter below 2.95 mm have been observed to have lower patency (Martinez-Mier et al., 2023). Ultrasound mapping is a method being explored to ensure fistula patency alongside these selection parameters and has been linked with better outcomes (Chlorogiannis et al., 2023).

### 3.4 Blood flow parameters

Previous studies have shown that several flow parameters, such as flow pattern, WSS, flow velocity, rate, volume, vorticity, pulsatility, and resistance index, all influence the maturation and patency of AVFs and AVGs, due to their connection with IH or stenosis. Among these, flow patterns (Figure 2) and WSS are the major players in determining the patency and the development of IH. Extreme high/low or oscillating WSS is caused by turbulent flow, while laminar flow shows relatively uniform, stable WSS with magnitudes in the physiological range throughout the anastomosis and connected vein. However, the ideal WSS level AVF maturation remains unclear. Cunnane et al. (2017), a review of hemodynamic factors contributing to vascular access dysfunction, found a multitude of papers in support of the link between pathologically high, low, or oscillating WSS with IH development (McNally et al., 2018). However, Northrup et al. (2022) indicated that high WSS, vorticity, and venous velocity on Day 1 (immediately post-operation) predicted successful AVF maturation. Kim et al. (2021) determined, however, that high WSS in the vicinity of the anastomosis of AVG was advantageous but became disadvantageous further away. Pike (2021) studied IH-prone locations in the context of AVG and found these locations showed high WSS and high circumferential stretch. This was linked to lower luminal remodeling in a pig model, thus indicative of endothelial dysfunction. In the presence of IH (after initial onset), there is a positive feedback loop–with worsening severity of IH, WSS kept elevated, and so does the oscillatory index (Zhu and Sakai, 2021).

Other flow parameters also possess prognostic functions for patency. Kudze et al. (2020) found that lower blood velocity was linked to diminished patency and development of IH. Abreu (2022) and Hammes et al. (2021) corroborated these findings via Doppler ultrasound and CFD modeling, respectively. Abreu (2022) further linked stenosis to both high resistive index and reduced pulsatility index from a preoperative high level to a postoperative low level. These studies highlight the importance of blood velocity: low velocity does not correctly “fight” the forming stenosis, while high blood velocity tends to centralize WSS causing inflammation.

### 3.5 Cellular mechanisms

The increased blood flow from the artery into the vein exposes venous VEC to elevated WSS, which triggers a cascade of molecular signaling pathways leading to inflammation and developing IH, causing failure of AVFs. Endothelial reaction to WSS causes the internal wall to thicken and thus eventually causes stenosis or thrombosis (Rai and Agrawal, 2022). There are several key signaling factors including Akt1-mTORC1 and endothelial nitric oxide synthase (eNOS), involved in the AVF maturation and patency of AVF or AVG.

Akt1-mTORC1 is a signaling molecule involved in cell growth and proliferation. In the context of AVFs, the over-expression of Akt1-mTORC1 is linked to IH development. Guo et al. (2019) determined that patient treatment with Rapamycin, an Akt1-mTORC1 inhibitor, in the initial stages of AVF formation reduced both inflammation and wall thickening. Though prevention of inflammation is beneficial, wall thickening is necessary for the vein to become strong enough for hemodialysis. Thereby, the Akt1-mTORC1 concentration must be controlled, not eliminated, for the successful AVF maturation, long-term access patency, and remodeling of the vein downstream to the AVG, the location prone to IH and stenosis.

Like Akt1-mTORC1, the eNOS signaling molecule must be regulated. It reduces wall thickening and prevents IH due to the key roles of nitric oxide in controlling endothelial dysfunction and blood flow regulation VSMCs. Pike et al. (2019) investigated the effects of NOS3 and by extension eNOS overexpression in mice. Overexpression of eNOS was linked to increased lumen diameter and thus smoother blood flow and lower vorticity; on the other hand, it was also linked to wall thinning due to reduced WSS and inner wall circumferential stress, both of which resulted from larger lumen. Kudze et al. (2020), Bai et al. (2022), and Somarathna et al. (2020) all supported the key role of eNOS in successful AVF maturation and IH prevention.

Besides endothelial reaction, subsequent responses from macrophage and VSMC (vasoactivity and remodeling) determine the long-term fate of access. Macrophages are immune cells that largely determine the access fate after inflammation. Macrophages are broadly classified into two phenotypes: M1 (pro-inflammatory) and M2 (anti-inflammatory and tissue-repairing). The former is involved in the initial immune response, but their prolonged presence can contribute to chronic inflammation, and thus tissue damage and fibrosis. In contrast, M2 macrophages are associated with tissue remodeling and angiogenesis for functional restoration (Mills, 2015). Therefore, the M1-to-M2 transition is of great importance to the healing process of anastomosis and the success of AVF (Samra et al., 2022). An efficient transition helps to dampen the initial pro-inflammatory response and facilitate tissue repair and graft integration meanwhile preventing fibrosis.

### 3.6 Cannulation-related complications

Cannulation issues are related to needle puncture-caused damage. Both AVFs and AVGs require repeated needle punctures for hemodialysis (Figure 1G), typically twice per session and at least 312 times annually. This repetitive cannulation leads to damage to the wall AVF and AVG including vascular endothelium (Hudson et al., 2019; Kumbar, 2012). This severely influences or even halts hemodialysis, particularly during the initial period (first 4–6 weeks) of cannulation, leading to complications such as hematoma formation, needle infiltration, and extravasation of blood at the cannulation site (Kumbar, 2012). In the inner layers of both AVFs and AVGs, large needle-induced scars are evident, demonstrating that the healing process of vascular endothelium trauma is prolonged, as observed in the findings of Donnelly and Marticorena (2012). In more severe instances, they can result in thrombosis, flow blockage, and irreversible access loss due to compression of the fistula lumen (Kumbar, 2012).

**FIGURE 4:**
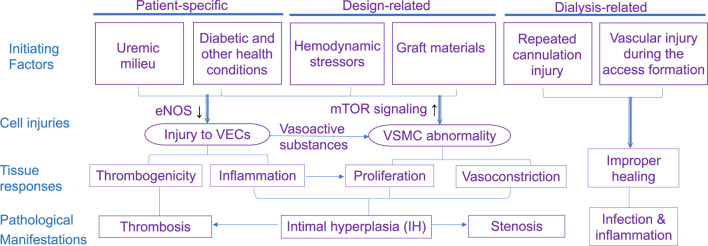
Mapping of various initiating factors leading to pathology in the dialysis access.

In conclusion, the management of vascular access in hemodialysis is fraught with various complications and pathologies, significantly impacting patients’ outcomes. Besides what is discussed above, a patient’s health conditions (Vazquez-Padron et al., 2021; Kingsmore et al., 2021) can also play a role. Therefore, an ideal access will be different for every hemodialysis patient. The intricacies of managing complications and pathologies underline the need for careful selection and maintenance of vascular access, as well as ongoing innovations in biomaterials and techniques to enhance the functionality and longevity of these methods. Figure 4 summarizes the factors contributing to the pathology of dialysis access.

## 4 Recent advancement in vascular access: technique, materials and device innovations

**FIGURE 5:**
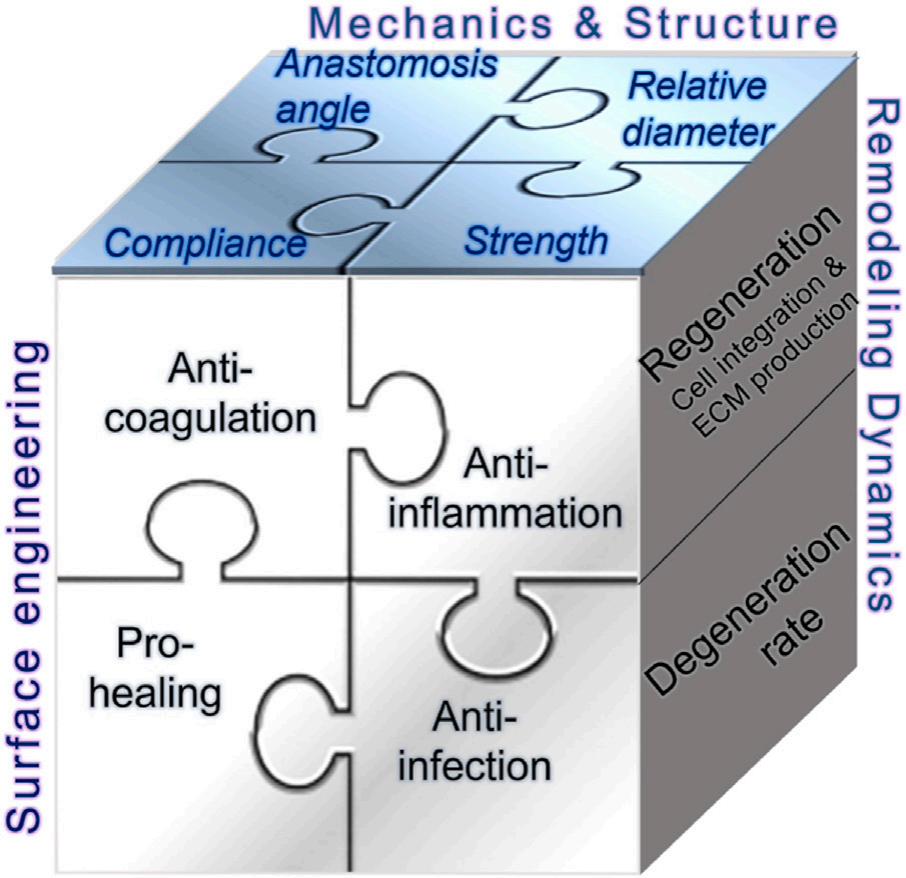
Illustrations of consideration pieces over innovations in vascular access.

As concluded in Section 2, developing technologies to enhance the performance of each access method may be necessary to cater to all needs. This section consolidates information about recent advancements in materials and devices, which have been or are undergoing preclinical studies or clinical trials. In particular, recent clinical trials have been instrumental in shaping the current landscape of vascular access. Key areas of focus include evaluating regenerative approaches, new pharmaceuticals, and various geometrical designs of AVGs. In recent years, the technology underpinning vascular access devices has undergone considerable evolution, with notable implications for hemodialysis treatments. This progression underscores a concerted push towards optimizing the patient experience, diminishing the incidence of complications, and prolonging the functional lifespan of these essential devices. Figure 5 summarizes current efforts in three main areas of improving vascular access–surface engineering, mechanics and structure, and remodeling dynamics. They, separately or in combination, may address significant issues underlying the dysfunction of vascular access, in particular AVG.

### 4.1 Cellular and regenerative approaches

Cellular and regenerative approaches involve using tissue engineering principles to develop functional blood vessel-like grafts as durable vascular access (Devillard and Marquette, 2021). The cell types often used are vascular cells either primary cells (i.e., autologous), mainly VEC and/or VSMC, or cells derived from stem/progenitor cells (Jover et al., 2018). Key techniques and concepts developed in the last 3 decades include (i) pro-regenerative scaffolds from synthetic materials or decellularization (Bejleri and Davis, 2019); (ii) seeding of autologous or allogeneic cells seeded onto a scaffold for the formation of a vessel-like structure; (Zou et al., 2016); (iii) bioreactor culture with mimetic physiological environments for cell growth and maturation into functional tissues with sufficient mechanical strength and vessel-like properties; (Moysidou et al., 2021); and (iv) implantation and integration (Zhao et al., 2013). As an example of this 4-step approach, recent clinical trials by Humacyte use acellular matrix technology to form an AVG that is surgically implanted into patients (Gutowski et al., 2020). Earlier clinical trials with the L'Heureux approach also adopted these tissue engineering steps using autologous cells and biodegradable materials (Heureux et al., 2020). Recent preclinical studies have focused on the final step–understanding and modeling the integration of grafts with native tissues. Blum et al. (2022) combined a computational model and a lamb model to demonstrate two stages of such integration in tissue-engineered vascular grafts: the initial stenosis caused by inflammatory factors and cells for early neotissue formation, and after the scaffold degrades, mechano-mediated neotissue remodeling for vessel-like structure and reduced narrowing. It is worth noting that ongoing research in the field of regenerative medicine may lead to further advancements and refinements, but it might be challenging to incorporate geometric designs and coatings, as illustrated below.

### 4.2 Improved graft materials

There have been considerable advancements in the materials used for grafts and catheters. New materials with improved resistance to infection and thrombosis have been developed, aiming to enhance the performance and safety of vascular access devices. Innovations in graft materials, such as heparin coatings, aim to reduce thrombosis and IH, showing promise in improving graft outcomes (Parada et al., 2020; Dawson et al., 2024; Vachharajani et al., 2021). Applying biocompatible coatings or lining materials to the graft’s luminal surface can improve its hemocompatibility, reducing the risk of clot formation and platelet adhesion (Derakhshani et al., 2023). Some coatings also release anticoagulant or anti-inflammatory agents to enhance graft performance further (Ding et al., 2023). New materials with self-healing materials such as those from InnAVasc-Medical are also appealing to reduce infection and thrombosis, after repeated needle puncture and access (Gage et al., 2020; Gage et al., 2021).

### 4.3 Geometric designs of AVGs

**FIGURE 6:**
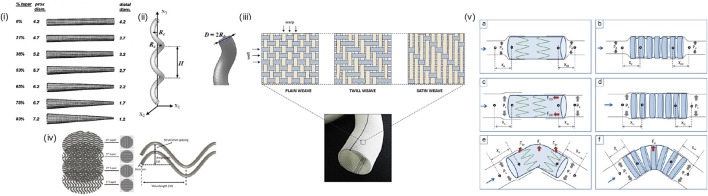
Geometric Design of AVGs: (i) tapered grafts (Lee et al., 2003), (ii) helical grafts (Ha et al., 2014), (iii) non-uniform luminal surface: structural design patterns of a woven Dacron^®^ graft. (Singh et al., 2015), (iv) waviness pattern (Ji and Guvendiren, 2020), (v) variable wall thickness: Schematic representation explaining the influence of compliance, columnar support, and SG curvature on migration intensity of Z-SG (left) and CaT-SG (right) during diastolic (a and b), and systolic (c–f) phase of fluid flow. (Xd, Xdd, Xs, and Xss denote the diastolic-proximal, diastolic-distal, systolic-proximal, and systolic-distal distance between SG and rubber sleeve neck, respectively. Pd, Ps, Fc, Fbr, and Fco denote diastolic pressure, systolic pressure, centrifugal force, bending-restoration force and columnar support force, respectively) (Singh and Wang, 2014).

Various geometrical designs of AVGs have been employed to enhance their biomechanical performance and long-term patency, through altering flow pattern and WSS to reduce intimal hyperplasia and thrombosis and/or to promote endothelial cell growth. Geometrical modifications include (Figure 6): (i) tapered grafts, which reduce the potential of disturbed flow at the anastomosis (Wiliams et al., 2021); (ii) curved, spiral, or helical grafts, which induce swirling or helical flow patterns, enhancing mechanical flexibility and thus reducing stress concentrations and disturbed (bidirectional) shear (Ene-Iordache and Remuzzi, 2017; Van Canneyt et al., 2013; De Nisco et al., 2020); (iii) non-uniform luminal surface modification, which include creating variations in the luminal surface texture such as grooves, ridges, or biomimetic patterns to influence the local hemodynamics and shear stress distribution; (Zizhou et al., 2022); (iv) waviness patterns: incorporating controlled waviness or sine wave patterns along the graft length can create controlled disturbances in blood flow (Moufarrej et al., 2016). This can promote healthy endothelial cell function and reduce the risk of stenosis (Jaideep et al., 2023); (v) variable wall thickness, which influences the mechanical properties of different graft segments and overall flexibility to help prevent kinking and bending while maintaining the structural integrity of the graft (Singh and Wang, 2014). It is important to note that while geometrical modifications hold promise for improving AVG performance, each approach comes with its challenges and considerations. Furthermore, the success of these modifications depends on factors such as the patient population, specific vascular conditions, and compatibility with existing surgical techniques. Researchers continue to explore and refine these geometrical modifications to optimize graft performance and increase their longevity in clinical applications.

### 4.4 Drug-coated stent grafts and balloons

**FIGURE 7:**
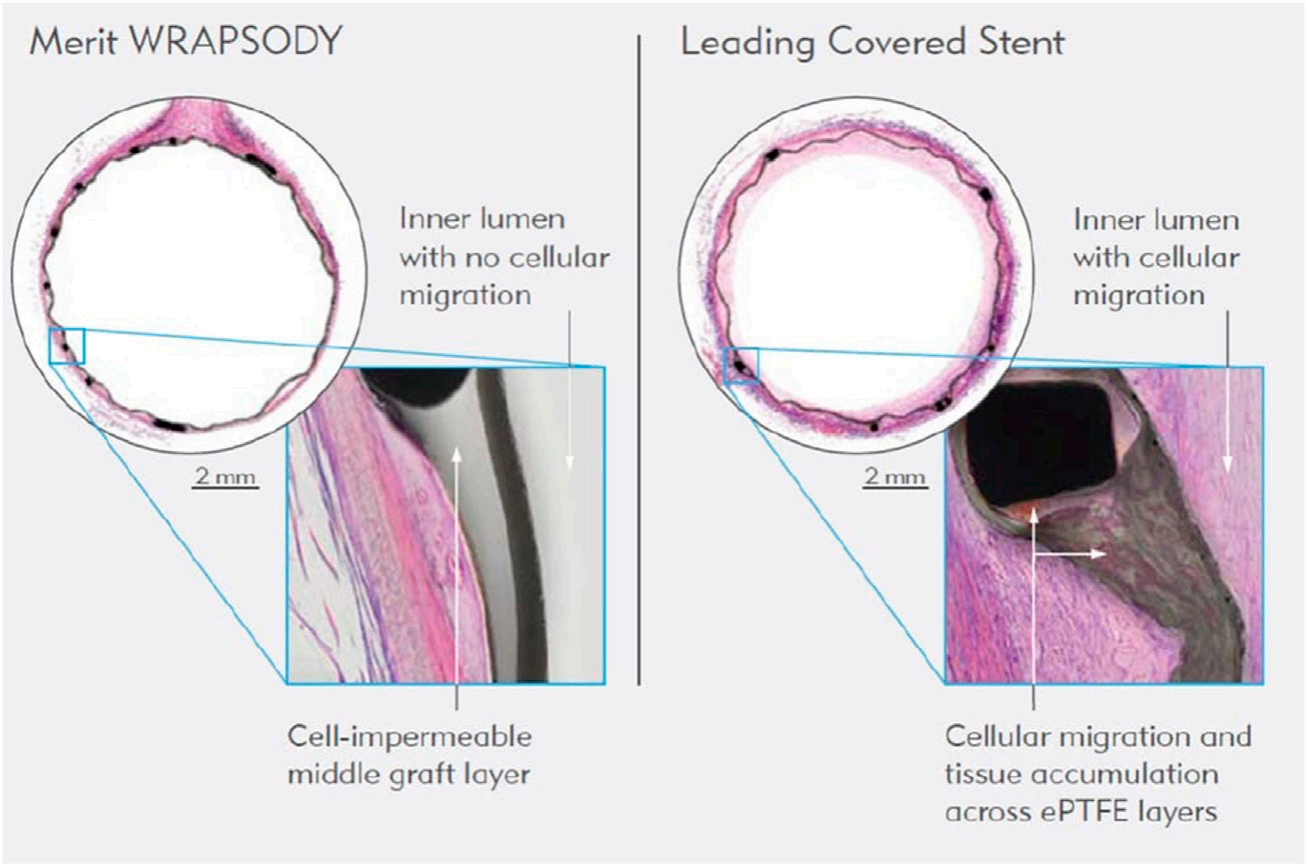
WRAPSODY™ Cell-Impermeable Endoprosthesis (https://www.merit.com/product/merit-wrapsody/?utm_campaign=socialmedia_2023Q4&utm_mediu m=infographic_wrapsody_1&utm_source=FB).

The introduction of drug-coated balloons (Figure 7) represents a significant advancement in the field (Ang et al., 2020). These devices, using local drug delivery technology, have shown efficacy in preventing IH and recurrence of stenosis, thus prolonging vascular access patency in hemodialysis patients (Vachharajani et al., 2021). Clinical trials comparing drug-coated to conventional balloon angioplasty have indicated a reduction in the number of interventions needed to maintain target lesion patency (Trerotola et al., 2018; Megaly et al., 2022).

### 4.5 Extravascular stents

Dai et al. (2021) have produced a new type of UV-curable HEMA stent encapsulating B. striata. The extravascular stent inhibits vascular expansion by providing mechanical support, and it releases B. striata to effectively limit the expansion of vein grafts inhibiting the formation of neointima. This study provides a solution for the restenosis of vein grafts after coronary artery bypass graft (CABG) and decisively suggests its success during its later clinical application.

### 4.6 Pharmaceutical trials and medication impact on AVG outcomes

Trials investigating the effectiveness of antiplatelet and antihypertensive medications, like clopidogrel (Ghorbani et al., 2009) and aspirin (Kaufman et al., 2003), have provided crucial insights into preventing primary AVG failure. Other important trials have examined the role of calcium channel blockers (Righetti et al., 2009) and angiotensin-converting enzyme inhibitors (Sajgure et al., 2007) in AVG maintenance. These studies are significant for understanding how medical treatments can complement physical vascular access methods to improve outcomes as well as guide clinical decisions regarding the management of vascular access in patients with ESKD (Lawson et al., 2020).

### 4.7 Innovative vascular access technologies

Additional research efforts have focused on novel vascular access technologies. For example, the recent trial involving the WRAPSODY™ Cell-Impermeable Endoprosthesis has demonstrated promising results in treating stenosis in arteriovenous access circuits, highlighting its potential to improve safety and effectiveness (Gilbert et al., 2021). Moreover, the development and clinical application of tissue-engineered vascular conduits shows potential for bioengineered grafts to be more durable and less prone to infection, stenosis, thrombosis, and aneurysms than conventional synthetic grafts, expanding options for patients with CKD (Vachharajani et al., 2021).

These clinical trials offer a comprehensive analysis of various aspects of vascular access, from the effectiveness of pharmacological interventions to the performance of new materials and techniques. The findings from these studies not only inform current practices but also pave the way for future innovations in the field.

## 5 Future direction

The evolution of vascular access strategies for hemodialysis is veering away from a rigid ‘fistula-first’ approach towards a more nuanced, patient-centered paradigm. This shift recognizes the complexities of arteriovenous fistula (AVF) maturation and the associated high rates of complications that have necessitated reevaluation of previous guidelines (Lok et al., 2020). Future strategies will likely focus on individualized treatment plans, acknowledging the diverse needs and health profiles of patients (Lok et al., 2020). This approach encompasses not only the type of vascular access but also considers the broader context of the patient’s kidney replacement therapy and life plan, emphasizing the need for regular updates and re-evaluations of these plans (Lok et al., 2020). As the field progresses, a balanced consideration of patient-specific factors, technological advancements, and emerging medical evidence will guide the development of more effective and less invasive vascular access options, thereby enhancing patient outcomes and quality of life in hemodialysis care.

## References

[B1] Abreu R. (2022). New hemodynamic variables as predictors of arteriovenous fistula maturation. Semin. Dial. 35 (4), 358–362. 10.1111/sdi.13062 35193155

[B2] Almasri J. Alsawas M. Mainou M. Mustafa R. A. Wang Z. Woo K. (2016). Outcomes of vascular access for hemodialysis: a systematic review and meta-analysis. J. Vasc. Surg. 64 (1), 236–243. 10.1016/j.jvs.2016.01.053 27345510

[B3] Ang H. Koppara T. R. Cassese S. Ng J. Joner M. Foin N. (2020). Drug-coated balloons: technical and clinical progress. Vasc. Med. 25 (6), 577–587. 10.1177/1358863x20927791 32634046

[B4] Asif A. Roy-Chaudhury P. Beathard G. A. (2006). Early arteriovenous fistula failure: a logical proposal for when and how to intervene. Clin. J. Am. Soc. Nephrol. CJASN 1 (2), 332–339. 10.2215/CJN.00850805 17699225

[B5] Bai H. Wei S. Xie B. Wang Z. Li M. Qiao Z. (2022). Endothelial nitric oxide synthase (eNOS) mediates neointimal thickness in arteriovenous fistulae with different anastomotic angles in rats. J. Vasc. Access 23 (3), 403–411. 10.1177/1129729821996537 33619996

[B91] Bharat A. Jaenicke M. Shenoy S. (2012). A novel technique of vascular anastomosis to prevent juxta-anastomotic stenosis following arteriovenous fistula creation. J. Vasc. Surg. 55 (1), 274–280. 10.1016/j.jvs.2011.07.090 22116048

[B92] Blum K. M. Zbinden J. C. Ramachandra A. B. Lindsey S. E. Szafron J. M. Reinhardt J. W. (2022). Tissue engineered vascular grafts transform into autologous neovessels capable of native function and growth. Commun. Med. 2, 3. 10.1038/s43856-021-00063-7 35603301 PMC9053249

[B6] Bejleri D. Davis M. E. (2019). Decellularized extracellular matrix materials for cardiac repair and regeneration. Adv. Healthc. Mater. 8, 1801217. 10.1002/adhm.201801217 PMC7654553 30714354

[B7] Bylsma L. C. Gage S. M. Reichert H. Dahl S. L. M. Lawson J. H. (2017). Arteriovenous fistulae for haemodialysis: a systematic review and meta-analysis of efficacy and safety outcomes. Eur. J. Vasc. endovascular Surg. official J. Eur. Soc. Vasc. Surg. 54 (4), 513–522. 10.1016/j.ejvs.2017.06.024 28843984

[B8] Carroll J. Varcoe R. L. Barber T. Simmons A. (2019). Reduction in anastomotic flow disturbance within a modified end-to-side arteriovenous fistula configuration: results of a computational flow dynamic model. Nephrol. Carlt. 24 (2), 245–251. 10.1111/nep.13219 29314372

[B9] Chlorogiannis D. D. Bousi S. E. Zachiotis M. Chlorogiannis A. Kyriakoulis I. Bellos I. (2023). Pre-operative ultrasound mapping before arteriovenous fistula formation: an updated systematic review and meta-analysis. J. Nephrol. 12 (22), 281–292. 10.1007/s40620-023-01814-6 PMC11043143 38133741

[B10] Cunnane C. V. Cunnane E. M. Walsh M. T. (2017). A review of the hemodynamic factors believed to contribute to vascular access dysfunction. Cardiovasc Eng. Tech. 8, 280–294. 10.1007/s13239-017-0307-0 28527110

[B11] Dai C. Chu T. Xiang Li Jiang H. Liu T. Yang Z. (2021). A novel UV-curable extravascular stent to prevent restenosis of venous grafts. Compos. Part B Eng. 225, 109260. 10.1016/j.compositesb.2021.109260

[B12] Dawson L. P. Rashid M. Dinh D. T. Brennan A. Bloom J. E. Biswas S. (2024). No-reflow prediction in acute coronary syndrome during percutaneous coronary intervention: the NORPACS risk score. Circ. Cardiovasc. Interv. 17, e013738. 10.1161/CIRCINTERVENTIONS.123.013738 38487882

[B13] De N. G. Gallo D. Siciliano K. Tasso P. Lodi R. M. Mazzi V. (2020). Hemodialysis arterio-venous graft design reducing the hemodynamic risk of vascular access dysfunction. J. Biomechanics 100, 109591. 10.1016/j.jbiomech.2019.109591 31902610

[B14] Derakhshani A. Saeedeh Hasani S. Navaei T. (2023). “Hemocompatible polymers for medical applications,” in Woodhead publishing series in biomaterials, handbook of polymers in medicine (Woodhead Publishing), 143–175. 10.1016/B978-0-12-823797-7.00005-8

[B15] Devillard Chloé D. Marquette Christophe A. (2021). Vascular tissue engineering: challenges and requirements for an ideal large scale blood vessel. Front. Bioeng. Biotechnol. 9, 721843. 10.3389/fbioe.2021.721843 34671597 PMC8522984

[B16] Ding K. Yu X. Wang D. Wang X. Li Q. (2023). Small diameter expanded polytetrafluoroethylene vascular graft with differentiated inner and outer biomacromolecules for collaborative endothelialization, anti-thrombogenicity, and anti-inflammation. Colloids Surfaces B Biointerfaces 229, 113449. 10.1016/j.colsurfb.2023.113449 37506438

[B17] Dixon B. S. Beck G. J. Vazquez M. A. Greenberg A. Delmez J. A. Allon M. (2009). Effect of dipyridamole plus aspirin on hemodialysis graft patency. N. Engl. J. Med. 360, 2191–2201. 10.1056/NEJMoa0805840 19458364 PMC3929400

[B18] Donnelly S. M. Marticorena R. M. (2012). When is a new fistula mature? The emerging science of fistula cannulation. Seminars Nephrol. 32 (6), 564–571. 10.1016/j.semnephrol.2012.10.008 23217337

[B19] Ene-Iordache B. Remuzzi A. (2017). Blood flow in idealized vascular access for hemodialysis: a review of computational studies. Cardiovasc Eng. Tech. 8, 295–312. 10.1007/s13239-017-0318-x 28664239

[B20] Franco R. P. (2021). Is the fistula first approach still valid? J. Bras. Nefrol. 43 (2), 263–268. 10.1590/2175-8239-JBN-2020-U001 33682871 PMC8257282

[B21] Gage S. M. Guziewicz M. Ilzecki M. Kazimierczak A. Kirkton R. D. (2020). Arterial reconstruction with human bioengineered acellular blood vessels in patients with peripheral arterial disease. J. Vasc. Surg. 72 (4), 1247–1258. 10.1016/j.jvs.2019.11.056 32093913

[B22] Gage S. M. Illig K. A. Ross J. R. (2021). Use of a novel immediate access dialysis graft designed to prevent needle-related complications: a first-in-man case report. J. Vasc. Access 22 (3), 475–479. 10.1177/1129729820917265 32370648 PMC7642106

[B23] Ghorbani A. Aalamshah M. Shahbazian H. Ehsanpour A. Aref A. (2009). Randomized controlled trial of clopidogrel to prevent primary arteriovenous fistula failure in hemodialysis patients. Indian J. Nephrol. 19 (2), 57–61. 10.4103/0971-4065.53323 20368925 PMC2847809

[B24] Gilbert J. Rai J. Kingsmore D. Skousen J. Ptohis N. (2021). First clinical results of the merit WRAPSODY™ cell-impermeable Endoprosthesis for treatment of access circuit stenosis in haemodialysis patients. Cardiovasc Interv. Radiol. 44, 1903–1913. 10.1007/s00270-021-02953-8 PMC8626397 34514534

[B25] Grechy L. Iori F. Corbett R. W. Gedroyc W. Duncan N. Caro C. G. (2017). The effect of arterial curvature on blood flow in arterio-venous fistulae: realistic Geometries and pulsatile flow. Cardiovasc Eng. Technol. 8 (3), 313–329. 10.1007/s13239-017-0321-2 28748414 PMC5573765

[B26] Guo X. Fereydooni A. Isaji T. Gorecka J. Liu S. Hu H. (2019). Inhibition of the akt1-mTORC1 Axis alters venous remodeling to improve arteriovenous fistula patency. Sci. Rep. 9, 11046. 10.1038/s41598-019-47542-5 31363142 PMC6667481

[B27] Gutowski P. Gage S. M. Guziewicz M. Ilzecki M. Kazimierczak A. Kirkton R. D. (2020). Arterial reconstruction with human bioengineered acellular blood vessels in patients with peripheral arterial disease. J. Vasc. Surg. 72 (4), 1247–1258. 10.1016/j.jvs.2019.11.056 32093913

[B28] Ha H. Hwang D. Choi W. R. Baek J. Lee S. J. (2014). Fluid-dynamic optimal design of helical vascular graft for stenotic disturbed flow. PLOS ONE 9 (10), e111047. 10.1371/journal.pone.0111047 25360705 PMC4215892

[B29] Hafeez M. S. Eslami M. H. Chaer R. A. Yuo T. H. (2023). “Comparing post-maturation outcomes of arteriovenous grafts and fistulae,” in The journal of vascular access, 11297298231151365 (Advanced online publication). 10.1177/11297298231151365 36847168

[B30] Halbert R. J. Nicholson G. Nordyke R. J. Pilgrim A. Niklason L. (2020). Patency of ePTFE arteriovenous graft placements in hemodialysis patients: systematic literature review and meta-analysis. Kidney360 1 (12), 1437–1446. 10.34067/KID.0003502020 35372887 PMC8815525

[B31] Hammes M. Moya-Rodriguez A. Bernstein C. Nathan S. Navuluri R. Basu A. (2021). Computational modeling of the cephalic arch predicts hemodynamic profiles in patients with brachiocephalic fistula access receiving hemodialysis. PLoS ONE 16, e0254016. 10.1371/journal.pone.0254016 34260609 PMC8279323

[B32] Hudson R. Johnson D. Viecelli A. (2019). Pathogenesis and prevention of vascular access failure. IntechOpen. 10.5772/intechopen.83525

[B90] Huber T. S. Berceli S. A. Scali S. T. Neal D. Anderson E. M. Allon M. (2021). Arteriovenous fistula maturation, functional patency, and intervention rates. JAMA Surg. 156 (12), 1111–1119. 10.1001/jamasurg.2021.4527 34550312 PMC8459303

[B33] Hull J. E. Balakin B. V. Kellerman B. M. Wrolstad D. K. (2013). Computational fluid dynamic evaluation of the side-to-side anastomosis for arteriovenous fistula. J. Vasc. Surg. 58 (1), 187–193.e1. 10.1016/j.jvs.2012.10.070 23433819

[B34] Jaideep A. Avinava R. Amit C. Gouripriya D. A. Sabu T. Manojit G. (2023). Effects of surface patterning and topography on the cellular functions of tissue-engineered scaffolds with special reference to 3D bioprinting. Biomater. Sci. 11, 1236–1269. 10.1039/D2BM01499H 36644788

[B35] Ji S. Murat G. (2020). 3D printed wavy scaffolds enhance mesenchymal stem cell osteogenesis. Micromachines 11 (1), 31. 10.3390/mi11010031 PMC7019315 31881771

[B36] Jover E. Fagnano M. Angelini G. Madeddu P. (2018). Cell sources for tissue engineering strategies to treat calcific valve disease. Front. Cardiovasc. Med. 5, 155. 10.3389/fcvm.2018.00155 30460245 PMC6232262

[B37] Kaufman J. S. O'Connor T. Z. Zhang J. H. Cronin R. E. Fiore L. D. Ganz M. B. (2003). Randomized controlled trial of clopidogrel plus aspirin to prevent hemodialysis access graft thrombosis. J. Am. Soc. Nephrol. JASN 14 (9), 2313–2321. 10.1097/01.asn.0000081661.10246.33 12937308

[B38] Kim J. T. Kim H. Ryou H. S. (2021). Hemodynamic analysis on the anastomosis angle in arteriovenous graft using multiphase blood model. Appl. Sci. 11 (17), 8160. 10.3390/app11178160

[B39] Kingsmore D. B. Stevenson K. S. Richarz S. Isaak A. Jackson A. Kasthuri R. (2021). Patient characteristics predict patency of early-cannulation arteriovenous grafts. Sci. Rep. 11, 10743. 10.1038/s41598-021-87750-6 34031434 PMC8144603

[B40] Krampf J. Agarwal R. Shenoy S. (2021). Contribution of inflow artery to observed flow in a vascular access: a computational fluid dynamic modeling study of an arteriovenous fistula circuit. J. Vasc. Access 22 (3), 417–423. 10.1177/1129729820944069 32729767

[B41] Kudze T. Ono S. Fereydooni A. Gonzalez L. Isaji T. Hu H. (2020). Altered hemodynamics during arteriovenous fistula remodeling leads to reduced fistula patency in female mice. JVS Vasc. Sci. 1, 42–56. 10.1016/j.jvssci.2020.03.001 32754721 PMC7402599

[B42] Kumbar L. (2012). Complications of arteriovenous fistulae: beyond venous stenosis. Adv. Chronic Kidney Dis. 19 (3), 195–201. 10.1053/j.ackd.2012.04.001 22578680

[B43] Lawson J. H. Niklason L. E. Roy-Chaudhury P. (2020). Challenges and novel therapies for vascular access in haemodialysis. Nat. Rev. Nephrol. 16, 586–602. 10.1038/s41581-020-0333-2 32839580 PMC8108319

[B44] Lawson J. H. Niklason L. E. Roy-Chaudhury P. (2020). Challenges and novel therapies for vascular access in haemodialysis. Nat. Rev. Nephrol. 16 (10), 586–602. 10.1038/s41581-020-0333-2 32839580 PMC8108319

[B45] Lee S.-W. Curi M. A. Baldwin Z. K. Balasubramanian V. Loth F. Schwartz L. B. (2003). Theoretical hydraulic consequences of vein graft taper. J. Vasc. Surg. 38 (4), 785–792. 10.1016/S0741-5214(03)00609-8 14560231

[B46] L'Heureux N. Labrunie G. Fénelon M. Dusserre N. Foulc M. P. Lafourcade M. (2020). Human textiles: a cell-synthesized yarn as a truly “bio” material for tissue engineering applications. Acta Biomater. 105 (2020), 111–120. 10.1016/j.actbio.2020.01.037 31996332

[B47] Li B. Abdelmasih M. Eisenberg N. Lok C. Roche-Nagle G. (2024). Long-term outcomes following thrombolysis of arteriovenous grafts. J. Vasc. Access 25 (3), 753–758. 10.1177/11297298211027470 34796766 PMC11075405

[B48] Li H. Jen S. Ramayya T. Bowers H. G. Rotem E. (2018). Unanticipated late maturation of an arteriovenous fistula after creation of separate graft access. Quantitative imaging Med. Surg. 8 (4), 444–446. 10.21037/qims.2018.01.03 PMC5989091 29928609

[B93] Liu P. He X. T. Zhang W. Fang Z. J. (2021). Analysis of patency rates and factors associated with arteriovenous fistula in maintenance hemodialysis patients followed for 10 years. Renal Failure 45 (2. 10.1080/0886022X.2023.2241923 PMC10512892 37724519

[B49] Lok C. E. Huber T. S. Lee T. Shenoy S. Yevzlin A. S. Abreo K. (2020). KDOQI clinical practice guideline for vascular access: 2019 update. Am. J. Kidney Dis. 75 (4), S1–S164. 10.1053/j.ajkd.2019.12.001 32778223

[B50] Martinez-Mier G. Cisneros-Tinoco M. A. Sanchez-Ruiz F. G. (2023). Vein and artery diameter influence on arteriovenous fistula maturation and patency in a Mexican population. J. Vasc. Access 24, 599–605. 10.1177/11297298211044023 34494490

[B51] McCullough K. P. Morgenstern H. Saran R. Herman W. H. Robinson B. M. (2019). Projecting ESRD incidence and prevalence in the United States through 2030. J. Am. Soc. Nephrol. 30, 127–135. 10.1681/asn.2018050531 30559143 PMC6317596

[B52] McNally A. Akingba A. G. Sucosky P. (2018). Effect of arteriovenous graft flow rate on vascular access hemodynamics in a novel modular anastomotic valve device. J. Vasc. Access 19 (5), 446–454. 10.1177/1129729818758229 30192183

[B53] Megaly M. Buda K. Saad M. Tawadros M. Elbadawi A. Basir M. (2022). Outcomes with drug-coated balloons vs. Drug-eluting stents in small-vessel coronary artery disease. Cardiovasc. Revascularization Med. 35, 76–82. 10.1016/j.carrev.2021.03.008 33858783

[B54] Mestres G. Gonzalo B. Mateos E. Yugueros X. Martínez-Rico C. Marcos L. (2019). Comparison of side-to-end vs. Side-to-Side proximal arteriovenous fistula anastomosis in chronic renal failure patients. Vascular 27 (6), 628–635. 10.1177/1708538119847392 31060450

[B55] Mills C. D. (2015). Anatomy of a discovery: m1 and m2 macrophages. Front. Immunol. 6, 212. 10.3389/fimmu.2015.00212 25999950 PMC4419847

[B56] Moufarrej A. Tordoir J. Mees B. (2016). Graft modification strategies to improve patency of prosthetic arteriovenous grafts for hemodialysis. J. Vasc. Access 17, S85–S90. 10.5301/jva.5000526 26951913

[B57] Moysidou C. M. Barberio C. Owens R. M. (2021). Advances in engineering human tissue models. Front. Bioeng. Biotechnol. 8, 620962. 10.3389/fbioe.2020.620962 33585419 PMC7877542

[B58] Murea M. Geary R. L. Davis R. P. Moossavi S. (2019). Vascular access for hemodialysis: a perpetual challenge. Seminars dialysis 32 (6), 527–534. 10.1111/sdi.12828 PMC6848759 31209966

[B59] Newby A. C. Zaltsman A. B. (2000). Molecular mechanisms in intimal hyperplasia. J. Pathol. 190 (3), 300–309. 10.1002/(SICI)1096-9896(200002)190:3<300:AID-PATH596>3.0.CO;2-I 10685064

[B60] Nojima T. Motomiya Y. (2021). Pathophysiology of high flow access and surgical flow reduction procedures. Kidney Dialysis 1 (1), 36–46. 10.3390/kidneydial1010007

[B61] Northrup H. He Y. Le H. Berceli S. A. Cheung A. K. Shiu Y.-T. (2022). Differential hemodynamics between arteriovenous fistulas with or without intervention before successful use. Front. Cardiovasc. Med. 9, 1001267. 10.3389/fcvm.2022.1001267 36407418 PMC9669082

[B62] Parada G. Yu Y. Riley W. Lojovich S. Tshikudi D. Ling Q. (2020). Ultrathin and robust hydrogel coatings on cardiovascular medical devices to mitigate thromboembolic and infectious complications. Adv. Healthc. Mater. 9, e2001116. 10.1002/adhm.202001116 32940970

[B63] Pike D. Shiu Y. T. Cho Y. F. Le H. Somarathna M. Isayeva T. (2019). The effect of endothelial nitric oxide synthase on the hemodynamics and wall mechanics in murine arteriovenous fistulas. Sci. Rep. 9, 4299. 10.1038/s41598-019-40683-7 30862797 PMC6414641

[B64] Pike D. B. (2021) “The effect of vascular mechanics on hemodialysis access remodeling in patients and animal models,”. Order No. 28721674 ed. Utah, United States: The University of Utah. Available at: https://colorado.idm.oclc.org/login?url=https://www.proquest.com/dissertations-theses/effect-vascular-mechanics-on-hemodialysis-access/docview/2737359116/se-2.

[B65] Rai V. Agrawal D. K. (2022). Transcriptional and epigenetic factors associated with early thrombosis of femoral artery involved in arteriovenous fistula. Proteomes 10, 14. 10.3390/proteomes10020014 35645372 PMC9149803

[B66] Remuzzi A. Bozzetto M. (2017). Biological and physical factors involved in the maturation of arteriovenous fistula for hemodialysis. Cardiovasc Eng. Technol. 8 (3), 273–279. Epub 2017 Jul 27. 10.1007/s13239-017-0323-0 28752375

[B67] Rezapour M. Sepehri M. M. Khavanin Zadeh M. Alborzi M. (2018). A new method to determine anastomosis angle configuration for arteriovenous fistula maturation. Med. J. Islam Repub. Iran. 32, 365–370. 10.14196/mjiri.32.62 PMC6325282 30643737

[B68] Righetti M. Ferrario G. Serbelloni P. Milani S. Tommasi A. (2009). Some old drugs improve late primary patency rate of native arteriovenous fistulas in hemodialysis patients. Ann. Vasc. Surg. 23 (4), 491–497. 10.1016/j.avsg.2008.08.033 18973987

[B69] Sajgure A. Choudhury A. Ahmed Z. Choudhury D. (2007). Angiotensin converting enzyme inhibitors maintain polytetrafluroethylene graft patency. Nephrol. dialysis, Transplant. official Publ. Eur. Dialysis Transpl. Assoc. - Eur. Ren. Assoc. 22 (5), 1390–1398. 10.1093/ndt/gfl821 17267534

[B70] Samra G. Rai V. Agrawal D. K. (2022). Heterogeneous population of immune cells associated with early thrombosis in arteriovenous fistula. J. Surg. Res. (Houst) 05, 423–434. 10.26502/jsr.10020237 PMC9354142 35937643

[B71] Shembekar S. N. Zodpe D. B. Padole P. M. (2022). Prediction of the anastomosis angle of arteriovenous fistula in hemodialysis to standardize the surgical technique. Biomed. Mater Eng. 33 (5), 423–436. 10.3233/BME-211389 35253728

[B72] Singh C. Wang X. (2014). A biomimetic approach for designing stent-graft structures: caterpillar cuticle as design model. J. Mech. Behav. Biomed. Mater. 30, 16–29. 10.1016/j.jmbbm.2013.10.014 24216309

[B73] Singh C. Wong C. S. Wang X. (2015). Medical textiles as vascular implants and their success to mimic natural arteries. J. Funct. Biomaterials 6 (3), 500–525. 10.3390/jfb6030500 PMC4598668 26133386

[B74] Somarathna M. Hwang P. T. Millican R. C. Alexander G. C. Isayeva-Waldrop T. Sherwood J. A. (2020). Nitric oxide-releasing nano matrix gel treatment inhibits venous intimal hyperplasia and improves vascular remodeling in a rodent arteriovenous fistula. Biomaterials 280, 121254. 10.1016/j.biomaterials.2021.121254 PMC8724452 34836683

[B75] Swaminathan S. Mor V. Mehrotra R. Trivedi A. (2012). Medicare's payment strategy for end-stage renal disease now embraces bundled payment and pay-for-performance to cut costs. Health Aff. Proj. Hope 31 (9), 2051–2058. 10.1377/hlthaff.2012.0368 PMC3766315 22949455

[B76] Thurlow J. S. Joshi M. Yan G. Norris K. C. Agodoa L. Y. Yuan C. M. (2021). Global epidemiology of end-stage kidney disease and disparities in kidney replacement therapy. Am. J. Nephrol. 52 (2), 98–107. 10.1159/000514550 33752206 PMC8057343

[B77] Trerotola S. O. Lawson J. Roy-Chaudhury P. Saad T. F. Lutonix AV Clinical Trial Investigators (2018). Drug-coated balloon angioplasty in failing av fistulas: a randomized controlled trial. Clin. J. Am. Soc. Nephrol. CJASN 13 (8), 1215–1224. 10.2215/CJN.14231217 30042225 PMC6086699

[B78] Vachharajani T. J. Taliercio J. J. Anvari E. (2021). New devices and technologies for hemodialysis vascular access: a review. Am. J. Kidney Dis. 78 (Issue 1), p116–p124. 10.1053/j.ajkd.2020.11.027 33965296

[B79] Van Canneyt K. Morbiducci U. Eloot S. De Santis G. Segers P. Verdonck P. (2013). A computational exploration of helical arterio-venous graft designs. J. Biomechanics 46 (2), 345–353. 10.1016/j.jbiomech.2012.10.027 23159095

[B80] Vazquez-Padron R. I. Duque J. C. Tabbara M. Salman L. H. Martinez L. (2021). Intimal hyperplasia and arteriovenous fistula failure: looking beyond size differences. Kidney360 2, 1360–1372. 10.34067/KID.0002022021 34765989 PMC8579754

[B81] Williams D. Leuthardt E. C. Genin G. M. Zayed M. (2021). Tailoring of arteriovenous graft-to-vein anastomosis angle to attenuate pathological flow fields. Sci. Rep. 11, 12153. 10.1038/s41598-021-90813-3 34108499 PMC8190231

[B82] Yang C.-Y. Li M. C. Lan C. W. Lee W. J. Lee C. J. Wu C. H. (2020). The anastomotic angle of hemodialysis arteriovenous fistula is associated with flow disturbance at the venous stenosis location on angiography. Front. Bioeng. Biotechnol. 8, 846. 10.3389/fbioe.2020.00846 32793578 PMC7390971

[B83] Zhao J. Qiu H. Chen D. Zhang W. Zhang D. Li M. (2013). Development of nanofibrous scaffolds for vascular tissue engineering. Int. J. Biol. Macromol. 56, 106–113. 10.1016/j.ijbiomac.2013.01.027 23384488

[B84] Zhou Yu Wu H. (2023). Comparison of end-to-side versus side-to-side anastomosis in upper limb arteriovenous fistula in hemodialysis patients: a systematic review and meta-analysis. Front. Surg. 9 (January 6), 1079291. 10.3389/fsurg.2022.1079291 36684232 PMC9853376

[B85] Zhu L. Sakai K. (2021). Simulation of blood flow past distal arteriovenous-graft anastomosis with intimal hyperplasia. Phys. Fluids 33 (5), 051905. 10.1063/5.0051517

[B86] Zizhou R. Wang X. Houshyar S. (2022). Review of polymeric biomimetic small-diameter vascular grafts to tackle intimal hyperplasia. ACS Omega 7 (26), 22125–22148. 10.1021/acsomega.2c01740 35811906 PMC9260943

[B87] Zou T. Fan J. Fartash A. Liu H. Fan Y. (2016). Cell-based strategies for vascular regeneration. J. Biomed. Mater Res. Part A 104A, 1297–1314. 10.1002/jbm.a.35660 26864677

